# Elastogenic Protein Expression of a Highly Elastic Murine Spinal Ligament: The Ligamentum Flavum

**DOI:** 10.1371/journal.pone.0038475

**Published:** 2012-06-07

**Authors:** Jeffrey P. Brown, Rachel M. Lind, Anthony F. Burzesi, Catherine K. Kuo

**Affiliations:** Department of Biomedical Engineering, Tufts University, Medford, Massachusetts, United States of America; University of Pittsburgh, United States of America

## Abstract

Spinal ligaments, such as the ligamentum flavum (LF), are prone to degeneration and iatrogenic injury that can lead to back pain and nerve dysfunction. Repair and regeneration strategies for these tissues are lacking, perhaps due to limited understanding of spinal ligament formation, the elaboration of its elastic fibers, maturation and homeostasis. Using immunohistochemistry and histology, we investigated murine LF elastogenesis and tissue formation from embryonic to mature postnatal stages. We characterized the spatiotemporal distribution of the key elastogenic proteins tropoelastin, fibrillin-1, fibulin-4 and lysyl oxidase. We found that elastogenesis begins *in utero* with the microfibril constituent fibrillin-1 staining intensely just before birth. Elastic fibers were first detected histologically at postnatal day (P) 7, the earliest stage at which tropoelastin and fibulin-4 stained intensely. From P7 to P28, elastic fibers grew in diameter and became straighter along the axis. The growth of elastic fibers coincided with intense staining of tropoelastin and fibulin-4 staining, possibly supporting a chaperone role for fibulin-4. These expression patterns correlated with reported skeletal and behavioral changes during murine development. This immunohistochemical characterization of elastogenesis of the LF will be useful for future studies investigating mechanisms for elastogenesis and developing new strategies for treatment or regeneration of spinal ligaments and other highly elastic tissues.

## Introduction

Lower back pain is the third most common cause for surgery in the U.S. [1], with stenosis in the lumbar spine being the primary reason for back surgery in the elderly [2]. Efforts to understand spine disease and improve treatments have focused primarily on the intervertebral disc, while minimal attention has focused on spinal ligaments, such as the ligamentum flavum (LF). Compared to other ligaments, the LF is an unusually elastic ligament due to its high proportion of elastic fibers. It acts as a passive stabilizing tissue, restoring the spine to a neutral posture following flexion and extension [3]. This spinal ligament is prone to calcification, ossification, and degenerative hypertrophy [4], and the resultant thickening of the tissue has been implicated as a significant cause of stenosis [4], [5]. Consequently, the affected back can suffer nerve impingement, radiating pain, and neurologic deficits. Unfortunately, treatment options are limited to surgical removal of the tissue without graft replacement. One challenge to developing new strategies to either treat diseased LF or regenerate new LF tissue is the limited knowledge we have about the development of this highly elastic tissue. Furthering our knowledge of how the LF forms may enhance the development of such needed strategies.

Elastic fibers, which are primarily composed of the protein elastin, impart resiliency to tissues and are vital to the function of several tissues in the body including the lungs, great vessels, and elastic ligaments [6]. The LF is composed principally of elastin and collagen, approximately in a 2∶1 ratio in humans [7]. Moreover, the LF has been found to have an elastin content of 40–60% in several mammals [8], including humans [7]. This compares to approximately 1% in skin, 28–32% in the aorta, 3–7% in the lung, and 4% in the Achilles tendon [9]. In destructive mechanical testing, a lacerated LF reduced the spine’s resistance to flexion by 25% [3]. *In vitro* studies revealed that the rupture strain for the LF averaged nearly 50% [7], as compared to 4–10% in the Achilles tendon [10], and 28% in the anterior cruciate ligament (ACL) [11]. While the unique mechanical properties and elastin content of the LF have been demonstrated, little attention has been given to the changes in tissue composition and structure that lead to such properties.

Elastogenesis requires the coordination of several proteins including tropoelastin (the soluble precursor to elastin), fibrillin-1 (fib-1), lysyl oxidase (LOX), and fibulin-4 (fbln-4) [12]. Fib-1 is a significant component of microfibrils [13], which are thought to act as a fiber scaffold for the deposition of tropoelastin because their appearance both precedes and then coincides with that of elastin during elastogenesis [14], [15]. LOX facilitates the crosslinking of tropoelastin into insoluble elastin [16] after proper coacervation and alignment of tropoelastin monomers [17]. Fbln-4 is less understood but is proposed to facilitate the deposition and/or crosslinking of tropoelastin [18], [19]. Much of the current knowledge of the roles these proteins serve in elastogenesis was gained through *in vitro* binding studies [20], [21] and knockout studies. Knocking out either elastin [22], LOX [23], fib-1 [24] or fbln-4 [19] caused disruptions to elastogenesis and perinatal death. What is lacking in the published works is characterization of the temporal hierarchy of elastogenic protein interactions *in vivo* during elastic fiber formation and tissue development.

We are interested in understanding the development and maturation of spinal ligaments, with a particular focus on the LF. Specifically, the aim of this study was to characterize the spatiotemporal expression of proteins integral to elastogenesis of murine LF during embryonic and postnatal development, skeletal maturation and aging. We characterized the spatiotemporal distribution and relative immunostaining intensity of tropoelastin, fib-1, LOX, fbln-4 and the corresponding formation of elastic fibers in murine LF tissues from embryonic day (E) 15 through postnatal day (P) 2-years.

## Methods

### Materials

All materials were from Sigma-Aldrich Co. (St. Louis, MO) unless otherwise specified.

### Tissue Harvest and Preparation

Scleraxis-green fluorescent (Scx-GFP) transgenic mice based on the C57BL/6 strain [25] were sacrificed by carbon dioxide asphyxiation and decapitation in strict accordance with the recommendations in the Guide for the Care and Use of Laboratory Animals of the National Institutes of Health. The protocol was approved by the Institutional Animal Care and Use Committee of Tufts University (Protocol Number M2011-23), and all efforts were made to minimize animal suffering. Spines were harvested from staged [26] embryos and mice (n ≥3) from E15 – 18 and P0, 7, 14, 21, 28, 35, 56 and 2-years (yrs). In postnatal mice, the lumbar region was dissected out from spines. Intact spine segments were fixed in methyl Carnoy’s solution and decalcified in isotonic 6% trichloroacetic acid. Spine segments were dehydrated by graded ethanol treatment, cleared in xylene, paraffin-embedded, and sectioned at 6 µm sagittally. Scx-GFP mice were originally chosen so that the fluorescent signal could be utilized to aid in identifying the LF; however, the GFP signal was severely diminished by tissue processing.

### Histology

Sections were deparaffinized in xylene, rehydrated, and stained with either hematoxylin and eosin (H&E) or Verhoeff Van Gieson (VVG). To visualize general tissue architecture and cellularity, sections were incubated in hematoxylin, differentiated in acid alcohol, and incubated briefly in eosin. To detect elastic fibers, collagen, and cell nuclei, sections were incubated in Verhoeff’s solution for 1 h, washed in tap water, differentiated in 0.25% w/v iron (III) chloride, and incubated briefly in Van Gieson solution. After staining, sections were rapidly dehydrated in graded ethanol washes, cleared in xylene, and mounted in DPX mounting medium (Electron Microscopy Sciences, Hatfield, PA). Sections from the sagittal plane running through the center of the LF tissue were selected for characterization and comparison.

### Immunohistochemistry

As previously described [27], sections were deparaffinized in xylene, rehydrated through ethanol gradient, permeabilized with 300 U/mL hyaluronidase and 0.025% Triton-X, treated with 5% H_2_O_2_ in methanol to neutralize endogenous peroxidase, and blocked for nonspecific binding using the Histostain-SP (Rabbit) kit (Invitrogen, Carlsbad, CA) blocking solution. Sections were incubated overnight with primary antibodies (tropoelastin (ab21601, 1∶100), fib-1 (ab53076, 1∶50), fbln-4 (ab74873, 1∶25) or LOX (ab31238, 1∶100), Abcam, Cambridge MA) or in primary antibody dilution buffer (phosphate buffered saline with 1% bovine serum albumin). Murine aorta and lung tissues were used as positive controls for all antibodies. The remainder of the immunohistochemical staining protocol was completed using the Histostain-SP (Rabbit) kit, following its instructions. Stained sections were dehydrated, cleared, mounted in DPX medium and visualized for brown DAB staining. Sections from the sagittal plane running through the center of the LF tissue were selected for characterization and comparison.

### Cellularity

**Figure 1:**
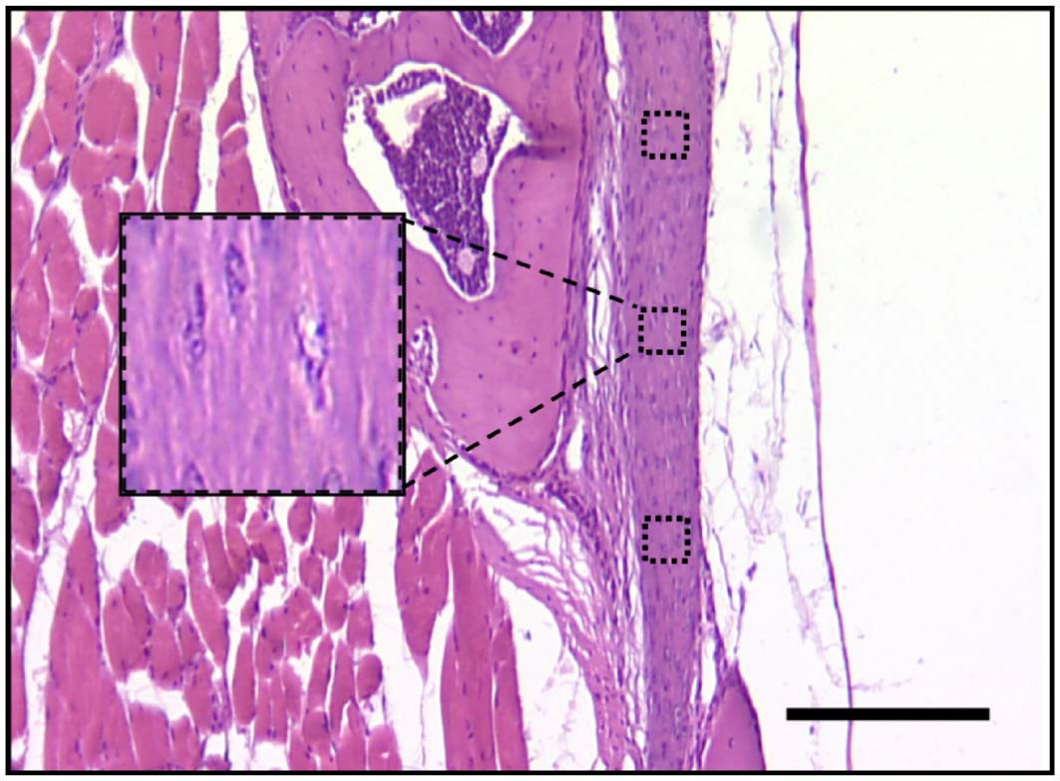
Determining cellularity of LF. Three representative 1000 µm^2^ areas of the LF were selected for cell counting at approximately equidistant regions along the length of the ligament. Counts were averaged for mice at a given stage for comparison (P56 shown). Scale bar: 200 µm.

H&E-stained sections of the LF from E16 through P2-yrs were imaged using an inverted optical microscope (Leitz Diavert, Wetzlar, Germany) and a DXC-390P color video camera (Sony, Tokyo, Japan). Three representative areas of the LF were selected for analysis at approximately three equidistant regions along the length of the ligament (**Fig. 1**). Stained nuclei in selected areas of a given LF were counted manually using ImageJ software (NIH, Bethesda, MD) and averaged for the animal. LF from ≥3 different animals were characterized for each stage. Means and standard deviations were calculated for all animals of a given stage or age.

### Statistical Analysis

Statistical significance of cellularity results was determined by the one-way analysis of variance using Dunnett’s posthoc test. *P*<0.05 was considered statistically significant.

## Results

### LF Tissue Morphology and Cellularity as a Function of Developmental Stage

**Figure 2:**
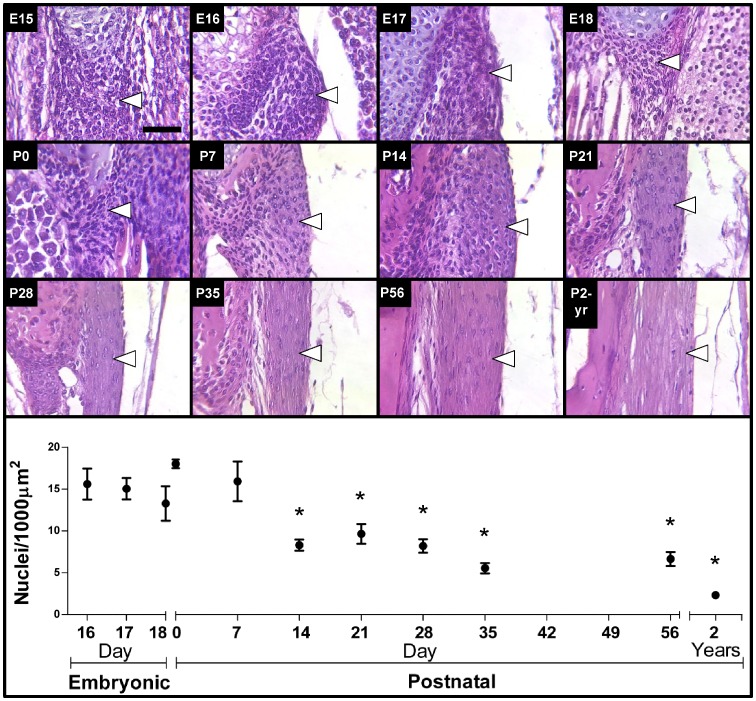
H&E staining and cellularity results of LF. *Top Three Rows:* Representative images of H&E-stained LF (arrows point just inside edge of tissue) from all stages characterized. Scale bar: 50 µm. *Bottom Panel:* Results of cell nuclei quantification in LF sections from E16 to P2-yrs embryos and mice. Cellularity of the LF shows a significant decrease at P14, and appeared to be lowest at P2-yrs. Mean ± standard deviations, *n* = 3. *P<0.05 versus P0.

Cellularity of the LF was characterized with H&E stained sections as a function of stage, ranging from E16 to P2-yrs (**Fig. 2** **)**. The overall trend observed was that cellularity of the LF was relatively high through P7, decreased by P14, and remained fairly steady through P56. Cellularity appeared to decrease to a minimum by P2-yrs. Cellularity at P0 was not significantly different from stages E16-E18 or from P7, however it was significantly higher than P14 and later ages. From P14 to P35, the LF developed a straighter and thinner morphology, with a rope-like appearance typical of adult tendons and ligaments.

### Elastic Fiber Formation

**Figure 3:**
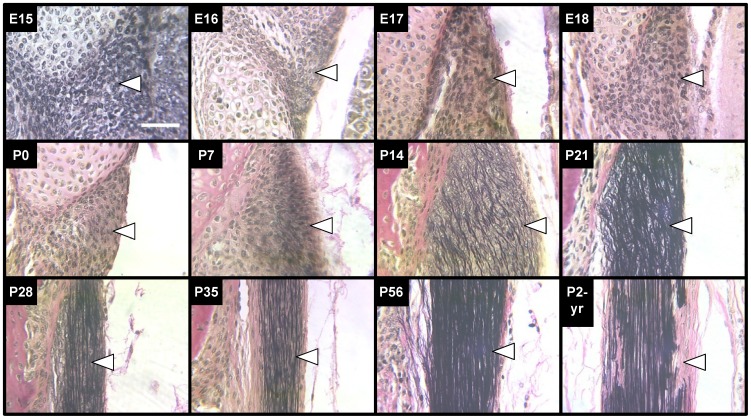
VVG staining of elastic fibers in LF during development, maturation and aging. VVG stains collagen (pink), nuclei (black) and elastic fibers (black). Elastic fibers of the LF (arrows point just inside edge of tissue) were first detected at P7 as thin and lacking orientation, but were thicker and aligned axially by P35. By P2-yrs, elastic fibers appeared fragmented in several areas. Scale bar: 50 µm.

Elastic fiber formation was characterized histologically as a function of embryonic and postnatal stages ranging from E15 to P2-yrs (**Fig. 3**). Based on VVG stained images, it appeared that the bulk of elastic fiber formation and apparent increase in elastic fiber diameter in the LF occurred during P0-P14. During late-embryonic and neonatal development, elastic fibers were not discernible. Elastic fibers were first visualized at P7, when they appeared thin and lacking organization. By P14, the elastic fibers appeared noticeably larger in diameter. Concurrent with the apparent increasing diameter and number of elastic fibers between P7 and P14, they appeared to become increasingly organized in a longitudinal orientation with a wavy or crimped shape. From P14 to P35, the elastic fibers appeared to become increasingly straighter along the longitudinal axis. Elastic fiber diameter and orientation did not appear to change at the ages studied beyond P35 (**Fig. 3**), although the density of fibers in the LF appeared to be greater at P56 than P35. By P2-yrs, the elastic fibers in the LF appeared to be fragmented in several areas of the tissue.

### Spatiotemporal Profiles of Elastogenic Proteins

#### Tropoelastin

**Figure 4:**
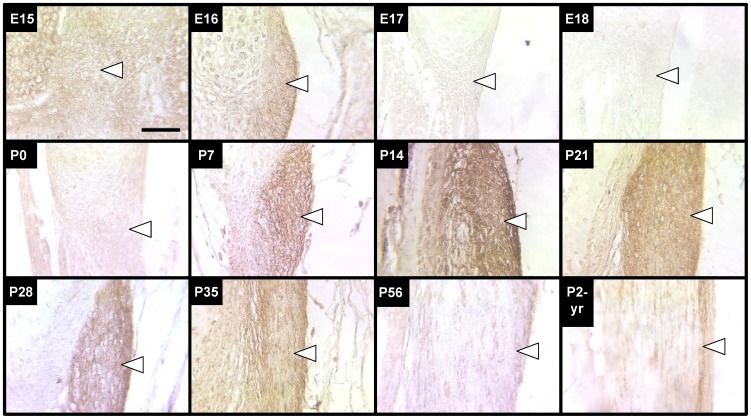
Tropoelastin immunostaining of LF during development, maturation and aging. Tropoelastin staining of LF (arrows point just inside edge of tissue) increased in intensity significantly first at P7 and remained high until P28. Staining intensity gradually decreased after P28 to a consistently low coloration that persisted throughout adulthood. Scale bar: 50 µm.

Tropoelastin staining was relatively weak in the LF from E15 through P0, with the exception of moderate staining intensity at E16 (**Fig. 4**). P7 was the first stage that staining intensity was observed to be substantially stronger, indicating a dramatic increase in tropoelastin production near this stage. Tropoelastin staining intensity remained relatively high until P35, when the staining intensity began to decrease. At P56 and P2-yrs, tropoelastin staining appeared minimal.

#### Fibrillin-1

**Figure 5:**
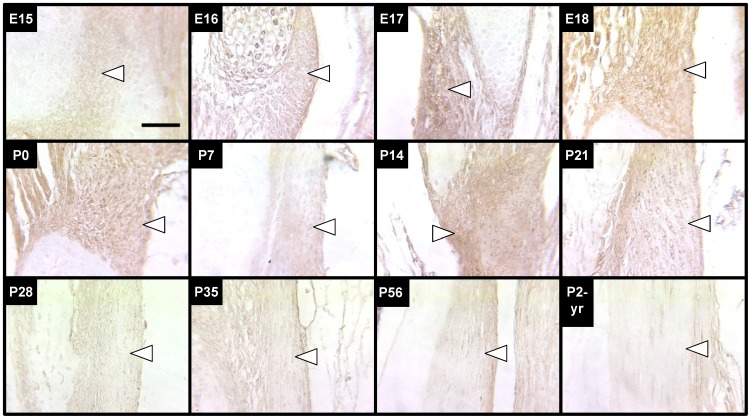
Fibrillin-1 immunostaining of LF during development, maturation and aging. Fibrillin-1 staining of LF (arrows point just inside edge of tissue) was most intense at E17. Staining intensity was high from E16 through P0 and at P14, but was less intense at P28 and older ages. Scale bar: 50 µm.

Fib-1 expression was intense in the LF from E16 through P0, with E17 appearing to be the stage with the greatest staining intensity (**Fig. 5**). Fib-1 stained with a moderate intensity from P14 through P21, but was markedly lighter at P28 and subsequent ages.

#### Fibulin-4

**Figure 6:**
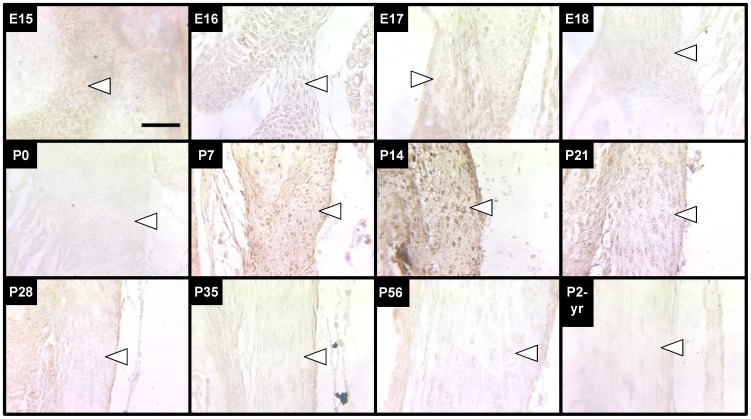
Fibulin-4 immunostaining of LF during development, maturation and aging. Fibulin-4 staining of LF (arrows point just inside edge of tissue) was high in intensity from P7 through P21, and was less intense at all other ages studied. Scale bar: 50 µm.

Fbln-4 staining intensity in the LF was greatest at P14, though moderately strong at P7 and P21 (**Fig. 6**). Before P7 and after P28, staining was detectable but relatively minimal.

#### Lysyl Oxidase

**Figure 7:**
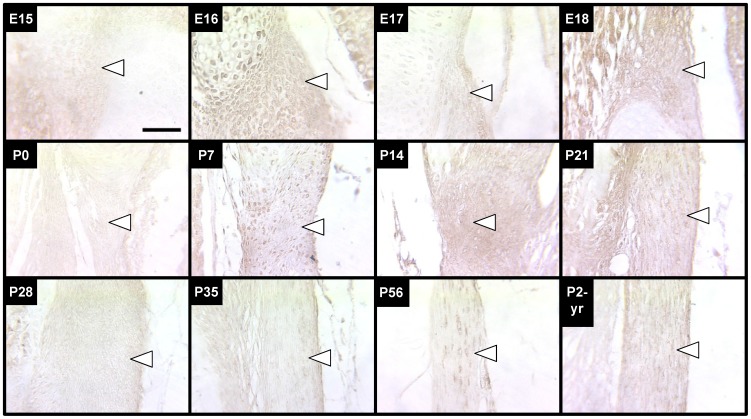
LOX staining of LF during development, maturation and aging. LOX staining of LF (arrows point just inside edge of tissue) was intermittently detectable through the stages studied, with higher staining intensities detected at E16, E18, and P7 through P21. Scale bar: 50 µm.

LOX staining intensity was intermittent in the LF over the stages studied (**Fig. 7**). Stages that stained intensely were E16 and E18, and P7 through P21. Additionally, light to moderate staining was observed at E17, P28, P56, and P2-yrs. Relatively little or no staining was observed at E15, P0 and P35.

## Discussion

**Figure 8:**
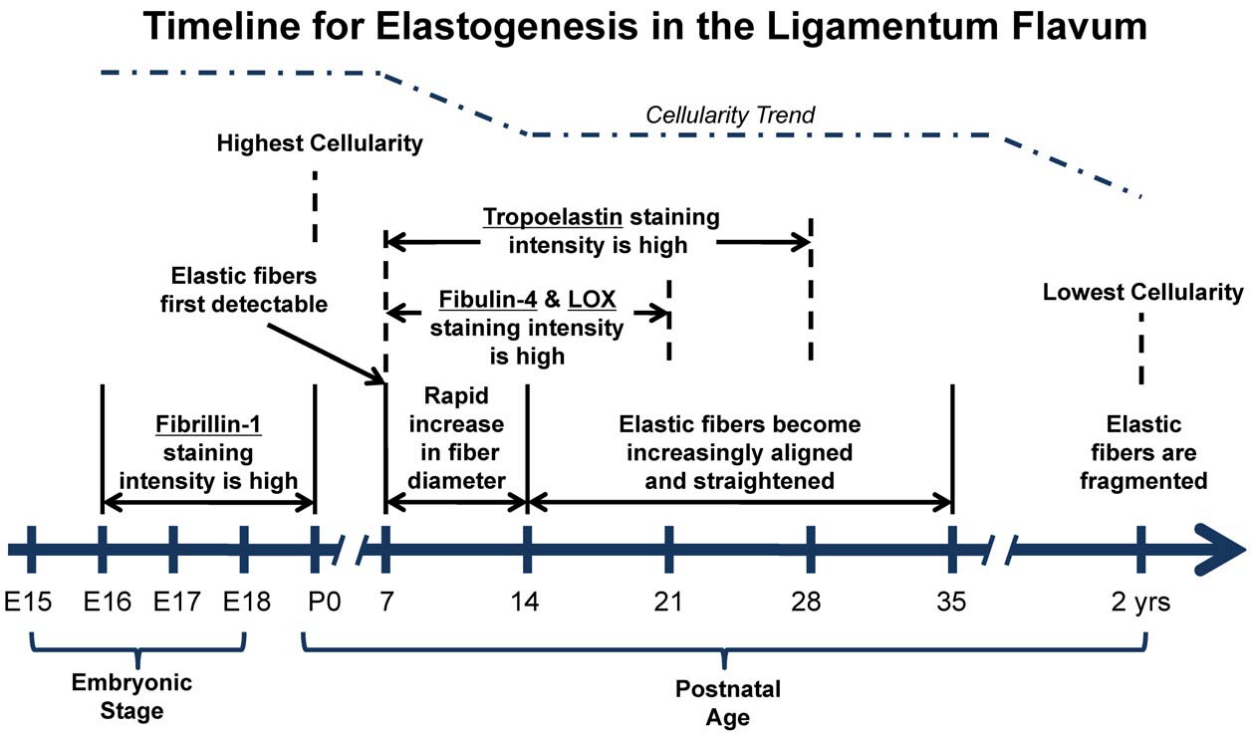
Timeline summarizing elastogenesis in LF during development, maturation and aging. Timeline summarizing elastogenic protein expression in the LF; stages/ages are in days unless otherwise indicated. Not drawn to scale.

Spinal ligaments are prone to various forms of degeneration that can lead to back pain and nerve dysfunction. Some spinal ligaments, such as the LF, are highly elastic. Little is known about how the LF develops beyond the observations that the embryonic LF is more cellular and has fewer elastic fibers than does adult LF [28], [29], which our data corroborates. Knowledge of the formation, homeostasis, and degeneration of the LF may enhance the development of repair and regeneration strategies for the LF. Toward that end, this study characterized spatiotemporal distribution of elastogenic proteins during LF development, maturation and aging (summarized in **Fig. 8**).

We first detected thin elastic fibers at P7. It is possible that very small elastic fibers were present in the LF prior to their first detectable appearance in bright-field images at P7. Previous studies using electron microscopy have shown that in the bovine nuchal ligament (NL), isolated elastic fibers first appear around mid-gestation and are below the resolution of light microscopy. These fibers begin as bundles of microfibrils that appear first around E90 (gestation 280 days), and by E135 they surround a small elastin core [15]. Through the rest of gestation, these core-sheath complexes increase in number and appear to fuse laterally, resulting in an increase in the elastin content and diameter of fibers [14]. The apparent earlier development and maturation of elastic fibers in the bovine NL may be related to its earlier functional requirements than that of the murine LF. Bovine calves are precocial and able to locomote immediately after birth. The mouse is advantageous as a model animal because it is relatively easy to breed, prolific, and because it is both mammalian and, unlike the calf, it is altricial like humans.

During maturation, we observed a unique straightening phenomenon in the elastic fibers of the LF that has not been reported in skin, aorta, or lung. In the LF, the apparent lack of organization of fibers at P7 was diminished by P14 when the elastic fibers instead appeared loosely crimped along the longitudinal axis. By P35 the fibers appeared to have lost their crimp and had straightened along the axis (summarized in **Fig. 8**). There are interesting correlations between the maturation of the LF elastic fibers and the timing of certain behavioral and physico-functional milestones in the mouse. On or about P7, the mouse can first walk in a straight line, stand on all four limbs, and normal posture is first attained [30]. P7 to P35 is the period of most rapid growth in the mouse [30], when the enthesis of the rotator cuff matures [31], and when the intervertebral disc completes much of its dimensional growth [32]. From a behavioral standpoint, this period is when mice can first perform very complex physical maneuvers, that nursing ceases, and that mice first leave their mothers in the wild [30]. Additionally, the apparent maturation of the LF and accompanying alignment and straightening of its elastic fibers by P35 may be related to the growth of other spinal tissues, and to the increase in magnitude and complexity of bodily movements leading up to that stage. Further investigation into the growth of spinal tissues or the strain regimen experienced by the LF during this period may be useful in identifying mechanisms for this phenomenon, and may warrant a future study.

Tropoelastin immunostaining intensity was greatest from P7 to P28 and diminished at later stages, though VVG histological staining indicated that mature elastic fibers persisted throughout adulthood. This is in agreement with previous studies that report that antibody binding of tropoelastin is greatly mitigated as it is crosslinked into insoluble elastin during tissue development [18], [33]. Presumably, this is due to a reduction in stereochemical availability of epitopes with increased crosslinking density of the fiber [12]. We speculate this may also be due to the chemical modification of lysine residues following deamination via LOX activity, but investigation of this mechanism was beyond the scope of this investigation.

Elastic fibers are thought to grow in diameter by the successive secretion, crosslinking, and incorporation of tropoelastin into insoluble fibers [14]. Our results support this insofar that tropoelastin was most abundant during the same period that we observed elastic fibers to grow demonstrably in apparent diameter (P7 to P14). Similarly, characterizations of the aorta, skin, and lungs have proposed and provided evidence to suggest that elastogenesis occurs during the embryonic or early postnatal period of development. Specifically, prior studies have found a spike in elastin gene expression in the mouse aorta and lung between E14 and P14 [12], [34], [35], indicating that for a variety of elastic tissues, elastogenic activity is heightened during the weeks surrounding birth. In contrast, there is little evidence of elastogenesis occurring in adult tissues [36]. One possible exception is skin, where studies suggest that elastogenesis is occurring in homeostatic adult tissues but that it appears erratic and disorganized [37]. Our immunostaining results suggest that elastic fiber assembly in the LF is primarily occurring during the first 4 weeks after birth, as evident by a visible increase in fiber diameter and the relatively abundant presence of soluble tropoelastin.

Fib-1 is a principal constituent of microfibrils, which are understood to act as the scaffold for elastic fiber formation. While mutations in fib-1 were found to be linked to Marfan Syndrome, an autosomal dominant disorder of connective tissue associated with vascular and pulmonary deficiencies [24], there is limited data available on fib-1 production in developing tissues. In the mouse aorta, fib-1 gene expression peaks at E16 and P0, then drops off steadily afterwards [34]. Our staining for fib-1 protein in the LF is consistent with this time period. Fib-1 is a structural constituent of elastic fibers, and the weak staining beyond P14 in our study, when elastic fibers were still present, seems likely due to the unavailability of epitopes for binding, as has been reported in other studies [12], [33]. Taken together, it appears that microfibril formation begins *in utero* in the mouse LF.

Recent studies have implicated fbln-4 in the regulation of tropoelastin expression, deposition, and crosslinking. In fbln-4−/− mice, elastic fibers are discontinuous and contain abnormal elastin aggregates [19], even though mRNA levels of tropoelastin and LOX are normal [19], [21]. However, the principal crosslink in elastic fibers, desmosine, is reduced by 85% in these mice [19]. When fbln-4 was knocked down *in vitro*, tropoelastin gene expression and protein deposition onto microfibrils were significantly reduced [18]. Moreover, when conditioned medium containing fbln-4 was added to cells haploinsufficient for the elastin gene, tropoelastin mRNA levels and deposition were significantly increased [18]. *In vitro* binding assays demonstrate that fbln-4 binds with proLOX and tropoelastin [21], but that such binding is inhibited by fib-1 [20], suggesting that fbln-4 may chaperone tropoelastin deposition and crosslinking by mediating interactions between these molecules [21]. The data presented in our study show tropoelastin and fbln-4 stained most intensely at the same stages of development, which is consistent with fbln-4′s purported role as a tropoelastin chaperone.

LOX is an enzyme critical to the development of extracellular matrix in connective tissues because it initiates the crosslinking of both collagen and elastin. Knocking out LOX in mice is perinatally lethal and results in dysfunction in elastic tissues, as well as fragmented elastic fibers [23]. Tropoelastin can form bi-, tri-, and tetra-functional crosslinks via LOX activity, and the degree of crosslinking may be regarded as a level of maturation of elastic fibers [38]. IHC staining of the rat aorta, which has elastic lamellae regionally intermixed with collagen, showed LOX expression was relatively strong through 18 weeks postnatal, but then decreased and was no longer detected in two year old animals [39]. Our study, however, shows an intermittent presence of LOX throughout embryonic and postnatal ages, including at two years of age.

Despite its importance to spine health and stability, the LF has garnered minimal attention, and the published studies are largely limited to Asian patients. It has been estimated that the prevalence of degenerated LF is approximately 5% in the Japanese [40] and 4% in the Chinese populations [41]. Such population studies are lacking in other demographics, despite reported case studies [42], [43], [44], [45]. In addition to the natural modes of degeneration that afflict the LF, there are also injuries to the LF that occur as a byproduct of the surgical process. The LF forms part of the smooth dorsal border of the spinal canal and it is resected routinely during several spine surgeries. This resection occurs without subsequent repair, despite cadaver studies demonstrating that cutting the LF reduces spine stability [3]. Moreover, the resulting fibrotic adhesions that can occur due to scarring have been cited as a leading cause for failed back surgery syndrome [46]. Scarring has been shown to alter the mechanical properties of ligaments [47], and therefore the iatrogenic injury has the potential to affect both the load bearing and the boundary functions of the LF. It has been proposed that some degenerative conditions in the spine are the consequence of biomechanical changes that resulted from injury or degeneration of a primary tissue [48], such as the disc or facet joint [49], which leads to biomechanical changes across the spine and subsequent degeneration in other tissues. A similar phenomenon has been observed following spinal fusion corrective surgery, where tissues at adjacent levels of the spine degenerate as a result of the altered biomechanics at the effected (primary) level [50]. Moreover, a related phenomenon has been observed in non-spinal joint tissues that also have multiple intimate points of contact [51]. Taken together, the codependent nature of connective tissues in the spine suggests that replacing or repairing the LF would be preferable to resection alone, which is the current treatment for symptomatic degenerating LF.

This is the first comprehensive descriptive characterization of a developing and maturing spinal ligament, and the first to provide spatiotemporal information about the changing cellularity and presentation of elastogenic proteins and elastic fibers during elastogenesis. These insights lay the groundwork for subsequent mechanistic studies to begin to elucidate molecular events involved in elastogenesis. In turn, our increased understanding may lead to treatments or improved repair and regeneration strategies for highly elastic ligaments that are critically important to musculoskeletal function.
